# WNT signaling – lung cancer is no exception

**DOI:** 10.1186/s12931-017-0650-6

**Published:** 2017-09-05

**Authors:** Judit Rapp, Luca Jaromi, Krisztian Kvell, Gyorgy Miskei, Judit E. Pongracz

**Affiliations:** 1 Department of Pharmaceutical Biotechnology, School of Pharmacy, University of Pecs, Pecs, Hungary; 2 Szentagothai Research Centre, University of Pecs, Pecs, Hungary

## Abstract

Since the initial discovery of the oncogenic activity of WNT ligands our understanding of the complex roles for WNT signaling pathways in lung cancers has increased substantially. In the current review, the various effects of activation and inhibition of the WNT signaling pathways are summarized in the context of lung carcinogenesis. Recent evidence regarding WNT ligand transport mechanisms, the role of WNT signaling in lung cancer angiogenesis and drug transporter regulation and the importance of microRNA and posttranscriptional regulation of WNT signaling are also reviewed.

## Background

Lung cancer (LC) is one of the deadliest forms of cancer worldwide [1, 2] affecting both genders [3, 4]. The two main types of LC-s are small cell lung cancer (SCLC) and non-small cell lung cancer (NSCLC). SCLC represents 15–20% of all LC cases and is the more aggressive form; it metastasizes early and therefore surgical intervention is rarely a therapeutic option [5]. On the other hand, NSCLC denotes 80–85% and can be further classified into adeno (AC)-, squamous cell (SCC) -, large cell (LCC) and various mixed type carcinomas [6]. Unfortunately, the majority of NSCLC patients are diagnosed at an advanced stage of the disease narrowing down therapeutic options and leading to a limited median survival of about 18 months [7]. Recent studies have confirmed that therapy-surviving cancer stem cells (CSC) play a cardinal role in drug resistance and therefore, rapid progression of the disease [8]. While the carcinogenic process in the lung can be traced back to genetic mutations, malfunctioning signaling pathways are also highly important modulators of tumor formation and individual features of the disease.

An increasing amount of evidence has shown that the WNT pathway is one of the main signaling pathways involved in maintaining lung homeostasis and that aberrant activation of this pathway may underlie several debilitating lung diseases. Similarly, to other human cancers, WNT signaling plays an important part in lung carcinogenesis. Interestingly, however, while some epigenetic changes that affect WNT pathway inhibitors are similar to those seen in other malignancies, genetic mutations of the WNT pathway are uncommon in NSCLCs [9].

This review will summarize some novel aspects of WNT signaling, what is currently known about WNT associated LC pathogenesis as well as some important features of WNT mediated events in LC therapies.

### The complexity of WNT signaling – Canonical and non-canonical WNT signaling pathways

**Fig. 1:**
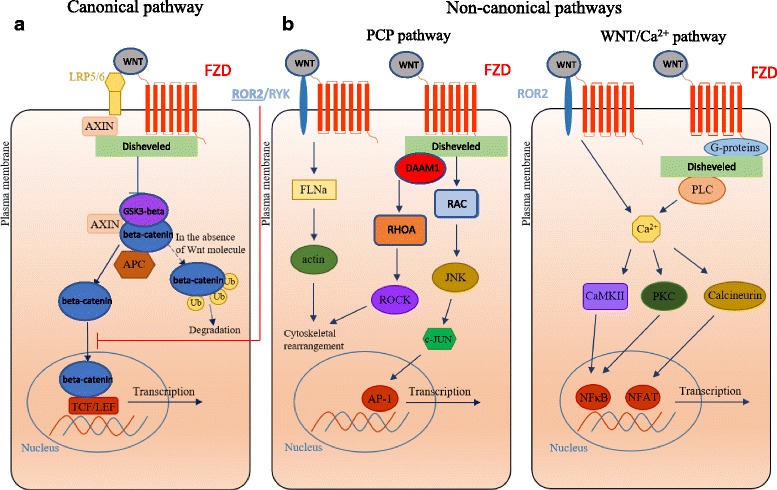
Multiplicity of canonical (**a**) and non-canonical (**b**) WNT pathways. Binding of WNT ligands to individual or different combination of their receptors including FZD and LRP5/6, or FZD in combination with ROR1, ROR2 or RYK activate multiple beta-catenin dependent (**a**) and beta-catenin-independent pathways (**b**)

WNT proteins are secreted glyco-lipoprotein morphogens that are required during lung development for cell-fate specification, cell proliferation and the control of asymmetric cell division. In adults, WNT signaling is essential for stem cell maintenance for regulation of tissue homeostasis [10]. Most of the 19 WNT ligands and the 10 main receptors, Frizzleds (FZD) that have been identified in mammalian cells can be identified in the human lung [9, 11]. The two main different WNT pathways include i) the beta-catenin-dependent or canonical pathway, and ii) the beta-catenin-independent or non-canonical pathways including the planar cell polarity (PCP) and the WNT/Ca2+ pathways (Fig. 1).

### Canonical or beta-catenin dependent WNT signaling.

In the lung, the role of WNT signaling has been examined in detail by multiple studies which mostly focus on beta-catenin-dependent signaling. In the canonical pathway during the absence of WNT, a beta-catenin destruction complex is assembled, consisting of: Axis inhibition protein (AXIN), adenomatous polyposis coli (APC), and glycogen synthase kinase 3-beta (GSK-3-beta) whereby beta-catenin is phosphorylated at serine and threonine sites and then proteolytically degraded [9, 12]. If WNT is available to bind to one of the ten FZD receptors then a receptor complex between WNT, FZD, lipoprotein receptor–related protein (LRP), Disheveled (DVL), and AXIN is formed [9]. Within this active complex, DVL becomes phosphorylated and eventually inhibits GSK-3-beta resulting in reduced phosphorylation and consequently stops the proteolytic destruction of beta-catenin. Beta-catenin subsequently accumulates in the cytoplasm. The cytoplasmic beta-catenin can then migrate to the nucleus and forms a complex with members of the T-cell factor (TCF)/Lymphoid enhancer-binding factor (LEF) family of transcription factors and transcriptional coactivators including cAMP response element-binding protein (CREB)–binding protein (CBP) and p300. The many target genes include c-myc and cyclin D1 [9]. The transmembrane receptor tyrosine kinase orphan receptor ROR2 (which is important in non-canonical WNT signaling) may also be involved in canonical signaling via interactions with FZD2 [13]. ROR2 [14], as well as the other WNT-binding receptors such as receptor-like tyrosine kinase RYK [15], can therefore act as regulatory receptors for the beta-catenin dependent WNT signaling.

#### Non-canonical WNT signaling

The two non-canonical WNT pathways are activated by several WNT ligands including WNT4, WNT5a, WNT7a, WNT11 and WNT16 [16–18]. Activation of the PCP signaling pathway, for example by WNT11, leads to the activation of the small GTPases RhoA (RAS homologue gene-family member A) and RAC1 (Ras-related C3 botulinum toxin substrate 1)**.** This, in turn, activates the stress kinases JNK (Jun N-terminal kinase) and ROCK (Rho-associated coiled-coil-containing protein kinase 1) that initiates remodeling of the cytoskeleton thus leading to changes in cell adhesion and motility [19–21].

The best known activator of Ca2 + −dependent WNT signaling is WNT5a. It triggers signal transduction via DVL-3, heterotrimeric G proteins and phospholipases [22]. Activation of this pathway leads to a transient increase in cytoplasmic free Ca2+ level that in turn can activate the protein kinase C (PKC) family, CaMKII (calcium calmodulin mediated kinase II) and the phosphatase calcineurin [23]. Apart from the well-known Ca2 + −dependent WNT signaling pathway, a novel, FYN tyrosine kinase and Signal Transducer and Activator of Transcription (STAT3) transcriptional regulator-mediated non-canonical WNT signaling pathway has also been identified in tumor cells [24].

Although WNT signaling pathways seem distinct, WNT proteins are promiscuous and can share receptors and regulate the expression of WNT signaling molecules, as well as modify WNT signaling pathway activity. Non-canonical WNT signaling, for example, represses canonical WNT activity via various mechanisms involving PKC-alpha, CaMKII-Transforming growth factor beta-Activated Kinase (TAK)1, Nemo-like Kinase (NLK), Siah2 E3 ubiquitin ligase or calcineurin-NFAT [21, 25–28]. Reports describing activation of the canonical pathway by non-canonical WNT ligands also exist [21, 29] making WNT signaling difficult to decipher and even more difficult to modulate in cancer therapy.

#### Functional variations of WNT signaling among cell types

Functional analyses of the canonical and non-canonical WNT pathways revealed that the canonical, PCP and Ca2+ pathways regulate multiple cellular activities in the lung that are dependent on the specific cellular context. In most cell types, non-canonical WNT signaling regulates canonical WNT activity, which is also critical for many aspects of lung biology. In response to canonical WNT signaling for example, beta-catenin/TCF/LEF signaling is activated in different lung cell types including the primordial epithelium (PE), alveolar epithelium (AE), and adjacent mesenchyme [30]. Human tissue studies have highlighted that in the developing lung, beta-catenin is found mainly in the peripheral epithelium, LEF1 expression is detected in alveolar and bronchial epithelium, while TCF4 is observed in epithelium and mesenchyme [31]. Tissue-specific deletion of beta-catenin in lung epithelial cells of test animals led to disrupted lung morphogenesis, lack of differentiation of the peripheral lung, enhanced formation of the conducting airways and consequently to death at birth due to respiratory failure [32]. Furthermore, beta-catenin phosphorylation can also lead to respiratory defects. Phosphorylation of beta-catenin at tyrosine 489 stimulates its nuclear localization and fibroblast activation which is a characteristic feature of bronchopulmonary dysplasia [33]. While deletion of the non-canonical WNT5a causes hyper-thickening of the mesenchymal interstitium and over-branching of the epithelial airways [34], overexpression of WNT5a in the epithelium disrupts epithelial-mesenchymal interaction and causes malformations in both the airway epithelium and the surrounding mesenchyme [35]. WNT5a also has a role in epithelial-mesenchymal transition (EMT) in LC; where expression of WNT5a and its receptor FZD2 have an inverse correlation with the expression of markers of epithelial differentiation, such as EpCAM, E-cadherin or keratin. Expressions of WNT5a and FZD2 positively correlate with the expression of vimentin, N-cadherin and fibronectin, which are well-known mesenchymal markers and are used to identify EMT during carcinogenesis [36].

Consequently, constitutive activation of either the canonical or the non-canonical WNT pathways in the developing lung can result in non-differentiated, dysfunctional lung phenotypes that resemble certain subtypes of LCs [37]. In support of this, investigation of constitutive activation of beta-catenin has shown that hyperactive canonical WNT signaling may channel NSCLC carcinogenesis towards the adenocarcinoma subtype [38].

### WNT signaling in LC

Various LC subtypes are believed to originate from stem cells in different histological parts of the lung. Adenocarcinoma, one of the NSCLC subtypes, has been reported to develop from various progenitor cells including alveolar type (AT) II cells, Clara cells, and bronchioalveolar stem cells (BASCs) [39–41]. The other NSCLC subtype, squamous cell carcinoma, initiates from basal cells [42] whereas SCLC is derived from pulmonary neuroendocrine cells (PNECs) [43]. Studies using genetic manipulation, however, have proved that such “histologically localized stem cell origin” approaches in LC are oversimplified. Overexpression of RAS for example in PNECs, a cell type thought to be the origin of SCLC leads to adenocarcinoma [44], while inactivation of p53 and Rb1 in ATII cells results in SCLC instead of adenocarcinoma [45]. Such studies indicate that driver mutations are more important than the cell of origin.

#### Murine cancer models

In murine models, activation of WNT signaling is associated with increased carcinogenic potential [46] especially if activation of canonical WNT signaling is triggered parallel with KRAS mutation [47, 48]. This process is also observed in human LC [49]. In human lung adenocarcinoma cases KRAS mutations are missense mutations which introduce amino acid substitution at one of the positions 12, 13, or 61. The result of these mutations is constitutive activation of the KRAS signaling pathway and it has been shown that if activation of KRAS and WNT signaling are combined, the joint activation leads to increased tumor size [49]. While tumors in WNT1 transgenic mice regress as WNT signals are blocked, tumor growth becomes WNT-independent in p53-deficient mice [50]. In the KRAS G12D substitution induced lung adenocarcinoma mouse model, WNT signaling enhances proliferation and EMT. Also, if down-regulation of SOX2 and upregulation of SOX9 and GATA6 simultaneously accompany KRAS mutation [51] then alterations in WNT signaling do not modulate the final outcome [48].

#### WNT pathway mutations differ in LC from other cancer types

The most studied WNT pathway mutations in cancers include inherited and sporadic mutations in APC and beta-catenin genes. Since APC is part of the degradation scaffold for beta-catenin, mutations of APC result in reduced degradation and increased nuclear accumulation of beta-catenin, leading to activation of target oncogenes including cyclin D1 and c-myc [52]. Such mutations are not universal in all cancer types and while APC mutations occur more frequently in cancers of the colon, the lung is rarely affected by such mutations. A constitutively active beta-catenin-LEF1 fusion protein under tissue specific promoter control has been designed to express mutant, degradation-resistant beta-catenin to mimic the effect of mutation in the degradation scaffold [37]. The fusion protein was used to mimic the constitutive activation of beta-catenin which has also been described in cancers of the lung. Increased levels of beta-catenin [53, 54] and loss of heterozygosity on chromosome 5q, which contains the APC locus, have been observed in LC types (Table 1). While specific site mutations of the APC [55] or the beta-catenin genes are rare in LCs, LC types are much better characterized by dysregulation of WNT ligand transcription [56–58]. For example, loss of WNT7a mRNA is a frequent feature of some LC cell lines and primary tumors [59]. NSCLC cells transformed with WNT7a show inhibition of anchorage independent growth via the JNK/AP1 dependent PCP signaling pathway [60]. In some other NSCLCs, elevated levels of WNT1 [61] and WNT2 [62] have been reported. Experimental inhibition of WNT2 induced signaling leads to down-regulation of the anti-apoptotic gene, Survivin and consequently initiates apoptosis [62]. The Sox2 gene coding the SOX2 transcription factor that is essential for maintaining self-renewal is also highly expressed in the main histological types of LCs [63]. Inhibition of SOX2 expression in lung adenocarcinoma induces apoptosis of tumor cells [64] and down-regulates WNT1/2, Notch1, and c-myc gene expression. On the other hand, stabilization of beta-catenin signaling blocks Clara cell differentiation to ciliated cells [65], while deletion of beta-catenin in basal cells is able to suppress proliferation and triggers apoptosis [66]. Moreover, autocrine insulin-like growth factor-I (IGF-I) signaling induces WNT5a dependent trans-differentiation of ATII cells to ATI-like cells [67].

**Table 1:** WNT ligands and signaling molecules associated with LC

Gene	Function	Mutation type/level of expression	References
APC	Part of beta-catenin destruction complex	No mutation Suppressed expression by promoter methylation	[184]
AXIN	Negative regulator of WNT signaling. Promotes beta-catenin phosphorylation which leads to beta-catenin degradation	No mutation Reduced expression	[185]
CTNNB1	Main component of canonical signaling, it serves as a transcription activator, binding to TCF/LEF family	Missense mutation of exon 3 results in substitution of Ser/Thr residues	[38]
		No mutation Increased expression in cytoplasm and nuclear compartment associated with poor prognosis	[186]
DKK1	Binding to LRP5/6 leads to its endocytosis and inhibition of canonical signaling	Increased expression detected in serum	[187]
DKK3	Secreted WNT antagonist	No mutation Reduced expression	[188]
DVL1	Required for FZD induced signaling pathway activation	No mutation Increased expression associated with advanced stages	[189]
DVL2		No mutation Increased expression is associated with advanced stages	[189]
DVL3		No mutation Increased expression	[190]
FZD8	Receptor for WNT proteins	No mutation Increased expression	[191]
GSK-3-beta	Phosphorylates beta-catenin resulting in beta-catenin degradation	No mutation Ser9 phosphorylation is associated with poor prognosis	[192]
SFRP1	Inhibits WNT signaling by binding to WNT proteins	No mutation Reduced expression regulated by promoter hypermethylation	[193]
TCF4	Transcription factor that complexes with beta-catenin upon activated canonical WNT signaling	No mutation Increased expression in poorly differentiated tumor	[194]
WIF1	Binding to WNT proteins to prevent their interaction with receptors	No mutation Increased expression	[195]
WNT1	Non-canonical WNT ligand	No mutation Increased expression in NSCLC	[196]
WNT11	WNT ligand which can activate both canonical and non-canonical WNT pathway	No mutation Increased expression	[68]
WNT2	Canonical WNT ligand	No mutation Increased expression	[197]
WNT3	Canonical WNT ligand	No mutation Increased expression	[107]
WNT5A	Non-canonical WNT ligand	No mutation Increased expression in SCC	[134]
WNT7A	Non-canonical WNT ligand	No mutation Reduced expression due to promoter hypermethylation	[198]
WNT7B	Canonical WNT ligand	No mutation Increased expression in AC	[199]

Most of the WNT pathway associated molecules are not mutated but the WNT signaling pathway is deregulated

#### Shifts between canonical and non-canonical WNT signaling modulate the carcinogenic process.

A shift from canonical to non-canonical WNT signaling, or vice versa, has also been reported in certain NSCLS subtypes. Up-regulation of the canonical WNT7b was detected in adenocarcinomas, while increased expression of WNT5a was found in primary squamous cell carcinomas [68]. Additionally, although the metastatic stage of any tumors are associated with EMT [69] and generally linked to increased beta-catenin-dependent signaling [70], the non-canonical WNT5a, which also regulates fibroblast growth factor (FGF) 10 and sonic hedgehog (SHH) expression [35], is overexpressed in lung metastases [71]. Matrix metalloproteinases, which are essential for tissue remodeling and are elevated in invasive cancers [72, 73], are target genes of both canonical and non-canonical WNT signaling pathways.

It is not only the WNT ligands, but also various signaling molecules that are dysregulated in LCs. For example, overexpression of DVL-3, a signal transducer molecule and positive regulator of WNT signaling pathways, was reported in 75% of primary NSCLCs compared to autologous matched normal tissue controls [74]. Down-regulation of WNT pathway antagonists, like Dickkopf-3 (DKK-3) [75], WNT inhibitory factor (WIF) [76, 77] and secreted Frizzled-Related Protein (sFRP) [78], have also been reported in various subtypes of LCs (summarized in Table 1).

#### Genome-wide association studies and LC susceptibility

Genome-wide association studies recently identified three LC susceptibility loci in chromosome regions 15q25, 5p15 and 6p21 [79]. Importantly, the nicotinic acetylcholine receptor (nAChR) subunit genes are located on the 15q25 chromosomal region. As nAChRs are expressed on bronchial cells and bind tobacco-related carcinogens with higher affinity than nicotine itself, therefore it is not surprising that the risk of LC is drastically increased in smokers [80]. Nicotinc AChRs in general, and alpha7 nAChRs in particular have been linked to nicotine-stimulated proliferation of lung carcinoma cells [81]. The nicotine induced up-regulation of WNT/PPAR-gamma (peroxisome proliferator activated receptor gamma) signaling can also regulate cigarette smoke-induced trans-differentiation of lung fibroblast to myofibroblasts that participate forming the cancer-associated stroma [82]. The BAT3 gene on the 6p21 locus affects p53 function and the cellular response to stress and apoptosis [83]. The same locus has also been associated with increased LC risk [80]. The telomerase reverse transcriptase (TERT) is located on the 5p15 locus and its modifications can cause aberrant proliferation and increased LC risk in both smokers and non-smokers [84]. The TERT-mediated developmental programs are similar to Myc and WNT-mediated responses and therefore, the increased risk for proliferative diseases is not surprising [84]. Interestingly, a genome-wide association study conducted on people who had never smoked revealed a strong correlation between the reduced transcription level of the glypican-5 (GPC5) gene and genotypes of the replicated SNP (rs2352028 at 13q31.3) in lung adenocarcinomas. GPC5 is a member of the glypican gene family of heparin sulphate proteoglycans that control the signaling pathway of WNT, hedgehog (HH), fibroblast growth factors (FGFs), and bone morphogenetic proteins (BMPs) which are all important regulators of cellular proliferation and differentiation [85].

### WNT ligands are “posted” in lipid envelopes

**Fig. 2:**
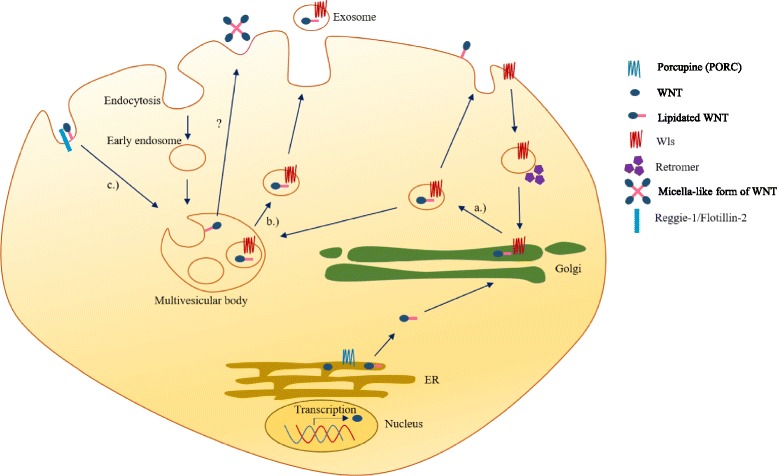
Mechanism of WNT secretion. WNT ligand is secreted in the endoplasmatic reticulum (ER) and palmitoylated by Porcupine (PORC). **a** After releasing from the ER palmitolyated WNT enters the Golgi apparatus and Wls-coupled WNT travels to the plasma membrane. Then Wls is recycled from the plasma membrane and the retromer complex shuttles it back to the Golgi. **b** WNT-Wls complex can be transferred to the microvesicular body. Exosome can be produced from MVB that serves as a source of WNTs for long-range spread. **c** Reggie-1/Flotillin-2 can mediate the re-endocytosis of WNT molecules and may facilitate the transport of a more soluble micella-like form of WNTs [89]

Gradients of WNT proteins are essential for tissue maintenance. Importantly, WNT gradients lead to different gene expressions at certain points of the gradient in the tissue. Such concentration differences generated along the gradient might even explain how designated “canonical” and “non-canonical” WNT proteins can alternate between signaling pathways. How the gradient is maintained is not yet entirely clear. Due to their lipid residues, WNT proteins are highly hydrophobic; they attach tightly to cell membranes [86] and then insert themselves into the lipid bilayer. If the lipid residues are removed from the amino acid backbone, WNT proteins become biologically inactive [87]. Due to their lipid modifications, WNT ligands understandably cannot be directly secreted into aqueous body fluids (Fig. 2). Recent studies indicate that WNT signals are most likely to be transduced via lipid-coated particles including extracellular vesicles [88]. For decades, extracellular vesicles or exosomes have been disregarded as a potential route of cellular communication, but recently they have leaped into the center of interest. Lipid envelopes can influence long-range WNT signal gradients [89] via Reggie-1/flotillin-2 (FLOT2) [90], a major component of lipid microdomains in membranes that can promote WNT secretion and diffusion [91]. Recent studies have revealed that high FLOT2 expression both mRNA and protein level predict poor outcomes in NSCLC [92]. Such findings indicate that it is not just differential WNT expression but also WNT concentration at various points of its gradient, membrane availability of WNT ligands and endocytosis of specific WNT molecules can change during carcinogenesis, thus modulating cellular activity and potentially drug response.

### Posttranslational modifications of WNT pathway molecules in regulation of LC

Secreted inhibitors and activators that regulate complex interactions within the complicated molecular network of WNT signaling [93] are under intense investigation. Epigenetic modulation, such as DNA methylation or histone deacetylation contributes to the deregulation of WNT signaling pathways. Down-regulation of several WNT signaling inhibitors have been reported in NSCLCs including AXIN*,* sFRPs 1–5*,* WIF-1*,* DKK-1*,* DKK-3*,* human homolog of Dapper (HDPR)1*,* runt-related transcription factor (RUNX)3*,* APC*,* caudal type homeobox (CDX)2*,* Dapper homolog (DACT)2*,* transmembrane protein (TMEM)88*,* Chibby*,* naked cuticle homolog (NKD1)*,* empty spiracles homeobox (EMX)2*,* inhibitor of growth family (ING)4*,* and miR-487b. Although the mechanisms are not yet entirely clear, methylation and hypermethylation are the likely causes of reduced inhibitor levels [94, 95]. DACT 2 is one of the Dact gene family members, which inhibit canonical WNT signaling. If expression of DACT2 is lost due to hypermethylation of its promoter, then beta-catenin dependent signaling is no longer suppressed and uncontrollable proliferation ensues [96]. Similarly to DACT2, GPC5 is downregulated in lung adenocarcinomas due to significant promoter hypermethylation. The methylation level of GPC5 promoter negatively correlates with its transcriptional expression and beta-catenin dependent signaling [97]. Restoration of its WNT pathway inhibitory function results in reduced WNT signaling, decreased cell proliferation, and increased apoptosis. Apart from promoter methylation of WNT pathway inhibitors, methylation of the beta-catenin promoter has also been described that leads to loss of beta-catenin protein expression and a poor prognosis in NSCLC patients [98].

Epigenetic modulation of the “anti-aging” Klotho [99] is also significant in carcinogenesis [100–102]. Klotho can act as an antagonist of the beta-catenin-dependent WNT signaling pathway; therefore overexpression of Klotho can reduce active beta-catenin and target c-myc and cyclin D1 levels [102], resulting in reduced proliferation. Supporting the above findings, down-regulation of Klotho increases cisplatin resistance while Klotho expression can attenuate resistance of LC to cisplatin-based chemotherapy and increase apoptosis [103]. Clinical survival analysis of various cancer types, however, has not demonstrated unequivocal involvement of Klotho in the carcinogenic process and it is still unclear whether the inconsistent role of Klotho in carcinogenesis is dependent on epigenetic variability or other factors [104, 105]. Recent discovery has exposed that apart from histone or DNA methylation of WNT signaling regulators, arginine methylation of the DVL-associated G3BP2 protein is a necessary post-translational modulation for LRP6 phosphorylation to initiate WNT3a induced canonical beta-catenin signaling from the receptor complex [106]. As WNT3a is one of the WNT ligands that promotes LC progression [107], investigation of methylation dependency of signaling molecules in the canonical signaling cascade can open up new therapeutic targets for drug discovery.

Methylation, however, is not the only posttranslational modification that modulates WNT signaling. AXIN, for example, can be destabilized by tankyrases [108] that regulate protein interactions and protein stability by poly-ADP-ribosylation. The post-translational modification of the N-terminal region of histone, by acetylation, methylation, ubiquitination, phosphorylation, or sumoylation, regulates DNA transcription, replication and repair. Recent epigenetic and transcriptomic profiling of human primary alveolar epithelial cells during in vitro differentiation revealed interactions amongst known regulatory pathways of distal alveolar epithelial cell differentiation. Interactions amongst the WNT signaling pathways, the transforming growth factor beta (TGF-beta) pathway, the hepatocyte nuclear factor 4 alpha (HNF4A) and the retinoid X receptor (RXR) signaling pathway [109] were strongly dependent on posttranslational changes.

### MicroRNAs targeting WNT signaling and LC

The non-coding microRNAs or miRNAs suppress gene expressions by inhibiting translation or increasing degradation of target gene mRNAs [110]. miRNAs, similarly to WNT proteins and various lipophilic molecules are delivered to target cells mostly in extracellular vesicles that are shed into body fluids from a great variety of cells for secure “message” delivery. For diagnostic purposes, collecting cancer specific miRNAs from extracellular vesicles rather than serum is more reliable, as the extracellular vesicle protects miRNAs from degradation [111].

Several miRNAs have been identified in association with various types of LCs in recent years (Table 2). For example, down-regulation of miR-29 and let-7 were shown to be DNMT3A⁄3B [112] and KRAS [113] targeting miRNAs, respectively, allowing upregulation of their targets. Meanwhile, KRAS, MYC, WNT5a, BMI1, and SUZ12 are targeted by miR-487b, which is down-regulated in certain types of LCs supporting the carcinogenic process [114]. Down-regulation of miR-214 levels was documented in cancer stem cells (CSCs) leading to stem cell marker expression including Nanog, OCT4, and SOX2 [115]. Amongst miR-214 targets in lung adenocarcinomas several beta-catenin-interacting proteins were also found [115], while beta-catenin itself was rather affected by miR-3619-5p. miR-3619-5p has been documented to suppress tumor growth in A549 and H460 NSCLC cell lines via binding to the 3′-UPR region of the beta-catenin gene [116]. Additionally, overexpression of miR-376c inhibits the growth of NSCLC cells via a WNT-related orphan nuclear receptor, the liver receptor homolog-1 (LRH-1) [117].

**Table 2:** miRNAs regulating WNT signaling in LC

microRNA	Regulation	References
miR-34a	Inhibits beta-catenin activity	[200]
miR-17-92	Increases beta-catenin activity	[201]
miR-21	Increases beta-catenin expression	[202]
miR-27b	Upregulated by WNT5a, inhibits vascular branching	[134]
miR-29	Downregulates beta-catenin expression	[203]
miR-31	Decreases WNT antagonists and increases WNT5a	[119]
miR-191	Increases beta-catenin pathway activation	[204]
miR-374a	Targets WNT5a	[205]
miR-376c	Suppresses canonical WNT signaling	[117]
miR-410	Activates beta-catenin pathway	[118]
miR-487b	Reduces WNT5a activity	[114]
miR-544a	Downregulates GSK3beta	[206]
miR-574-5p	Enhances beta-catenin phosphorlyation	[207]
miR-708	Increases canonical WNT signaling	[208]

In contrast, miR-410 can accelerate tumor growth by suppressing the expression of SLC34A2, a type 2b sodium-dependent phosphate transporter (NaPi-IIb) that is located in the apical membrane of ATII cells. Decreased NaPi-IIb levels have been shown to activate the WNT/beta-catenin pathway leading to enhanced tumor growth and invasion [118]. Perhaps not surprisingly, miRNA expression is also regulated by one of the primary causes of LCs, cigarette smoke. miR-31 for example that targets the WNT pathway inhibitor DKK1 is triggered by cigarette smoke leading to enhanced tumorigenesis in the lung [119].

Although some miRNAs have not been directly associated with WNT signaling in NSCLCs, in other tumor types they have been demonstrated to play important WNT pathway regulatory function. For example miR-148b suppresses tumor growth via inhibiting canonical WNT signaling in hepatocellular carcinoma [120], while miR-499 can stimulate blood vessel formation via WNT signaling activation in various other tumor types [121]. Their precise role in LCs awaits further investigation.

### WNT signaling in LC angiogenesis

WNT signaling has a fundamental role in both normal and tumor angiogenesis [122]. The canonical WNT pathway can regulate cadherin junctions in endothelial cell connections and therefore vascular permeability [123, 124]. Additionally, WNT signaling controls trans-endothelial migration of tumor cells via beta-catenin-dependent regulation of endothelial VE-, E- and N-cadherin expression [123–125]. The WNT-Ca2+ pathway -which is often referred to as a pro-angiogenic signaling pathway- induces endothelial cell proliferation and enhances capillary network formation, while activation of the PCP WNT pathway coordinates endothelial cell migration [126]. Activation of the PCP pathway via FZD4 impairs vascular morphogenesis [127], while activation of downstream components, such as DAAM-1 can reverse the changes [126]. The WNT/PCP pathway is responsible for impaired pericyte motility and as pericytes are important components of vessel formation and integrity, the balance of WNT pathways are important in forming and maintaining a functional lung vasculature [128]. Any imbalances in canonical and non-canonical WNT signaling could, therefore, modulate blood vessel formation in tumors and consequently affect therapeutic responses.

#### Induction of neovascularization – Similarities and differences in LC subtypes

Cancer cells can induce neovascularization when the solid tumor reaches more than 2 mm in diameter [129] and hypoxia occurs in the inner center of the tumor. Intra-tumor angiogenesis is best characterized by microvessel density. Extensive vascularization and higher vessel density indicate disease progression and predict a poor outcome in NSCLCs [130]. Amongst several other pro-angiogenic factors like Hypoxia-Inducible Factor (HIF)1alpha [131], Vascular Endothelial Growth Factor (VEGF)-A is of vital importance for endothelial cell proliferation and motility [132]. The pro-angiogenic factors are under complex molecular regulation. VEGF-A for example is documented to be under beta-catenin-dependent, canonical WNT control via a PPAR-gamma dependent mechanism [133]. Interestingly, PPAR-gamma down-regulation that is essential for VEGF-A up-regulation, can also be induced by a WNT5a triggered miR27b dependent manner [134]. Although tumors including NSCLC subtypes use the same molecular components to attract the necessary cell types for blood vessel formation as normal tissues, tumor vessels are often leaky, poorly differentiated and not hierarchic. This is due to differential WNT expression induced modulation of cellular morphology and function leading even to cellular mimicry of cells in the resident vascular network [135, 136]. For instance, WNT5a signaling can induce vascular mimicry [137], while canonical pathway activation by WNT3a or WNT7b is associated with increased angiogenesis [138, 139].

#### Angiogenesis and WNT target genes: Matrix metalloproteinases

The canonical WNT pathway mediated EMT [140], which correlates with E-cadherin down-regulation [68] and VEGF-A up-regulation is associated with micrometastasis formation [141, 142]. Blood vessel formation in lung tumors, however, also need the WNT target proteolytic matrix metalloproteinase enzymes (MMPs) [143] that are responsible for degradation of the extracellular matrix components during new blood vessel formation or vessel branching. MMP-2, −3, −7 and MMP-9 have been shown to be important in NSCLCs [93] and angiogenesis in general [143]. One of the most studied enzymes is MMP-9 which degrades type IV collagen, modulates VEGF bioavailability through direct cleavage and also regulates vascular permeability [144]. In lung adenocarcinomas, MMP-9 levels correlate with increased risk of relapse [143, 145], although its direct regulation by canonical WNT3a signaling has only been studied in colorectal cancer [146]. MMP-7 is up-regulated by canonical WNT signaling and associated with increased invasion of LCs [147–149]. Additionally, up-regulation of MMP1 by the non-canonical WNT5a has also been shown in NSCLC [137]. Proteomic analysis of an Mmp1−/− mouse model revealed that tumor growth is hampered by the absence of MMP1 activity and is also associated with decreased levels of chitinase-3 like 3 (CHI3L3) and accumulation of the receptor for advanced glycation end-products (RAGE) and its ligand, S100A8 [150]. The molecules identified in the mouse model are important markers for lung development, aging and tumorigenesis. Upregulation of RAGE, for example, is observed in type I alveolar epithelial cells (ATI) during lung development, which process is associated with reduced beta-catenin-dependent WNT signaling [151]. Interestingly, during aging RAGE is highly expressed [152], while in malignant lung tumors RAGE is down-regulated [153, 154]. More studies are needed to explain such differences and to identify the primary role of RAGE in aging normal lung tissue and in LC.

#### The angiogenic process as a therapeutic target

As the angiogenic process is a therapeutic target in solid tumors, anti-angiogenic agents have become important in therapy. The anti-VEGF-A monoclonal antibody, bevacizumab is the first approved antiangiogenic agent to be applied in NSCLSs [155]. Despite some success of anti-angiogenic agents in cancer treatment, the expected break-through in cancer therapy has not occurred. A randomized phase II study investigated the effect of bevacizumab mono- and paclitaxel-carboplatin-bevacizumab combination therapy. Although the latter combination increased progression free survival, serious hemorrhage was detected in some patients mostly with squamous histology [156]. As there is a well-documented difference in the WNT ligand profile of the two NSCLC subtypes [68], further studies of WNT controlled regulation of blood vessel formation is needed to more effectively stratify patients for the most appropriate anti-angiogenic treatment. The most worrying aspect is that in some cases – mostly in glioblastomas [157–160] - administration of anti-angiogenic drugs contributed to formation of even more invasive tumors and failure of cytotoxic treatment.

### Maintenance of cancer stem cells and therapeutic resistance

The accumulation of highly chemotherapy resistant cancer stem cells (CSC) are thought to play an important role in the incurability of LCs [161]. In CSCs beta-catenin dependent canonical WNT signaling is highly active and helps to maintain CSCs that express putative stem cell markers such as octamer-binding transcription factor 4 (OCT4) [162], Leucine-Rich Repeat-containing G-protein coupled receptor 5 (LGR5/GPR49), CD44, CD24, EpCAM [163], and cyclin D1 [11, 162]. The above markers are associated with increased cell proliferation rate and clone formation efficacy [164]. Such CSCs are highly resistant to several chemotherapeutic drugs [164] due to overexpression of ATP-binding cassette (ABC) transporter protein G2 (ABCG2 or BCRP1) [165, 166] resulting in increased drug efflux [167]. Apart from ABCG2 the presence of other ATP-binding cassette (ABC) transporter family members, such as ABCB1 (MDR1 or Pgp) are frequently tested as they are responsible for chemotherapeutic drug removal from cancer cells. Recently, the canonical WNT pathway dependent beta-catenin target TCF/LEF transcription factors were shown to activate the ABCB1 promoter indicating that WNT signaling is involved in the regulation of efflux transporter expression [168]. Studies of ABCB1 revealed that paclitaxel and irinotecan - the two drugs often used in cisplatin or carboplatin combination therapy of LCs [169, 170] - are both substrates of ABCB1. Cisplatin, a widely used drug in LC therapy, can also activate the canonical WNT pathway [162] which may explain the increased expression of efflux drug transporters including ABCB1 and ABCG2 correlating with reduced survival of NSCLC [171] and SCLC patients [172]. In contrast, inhibition of the beta-catenin dependent WNT pathway by high serum levels of the canonical pathway inhibitor DKK1 [173] has been detected in patients suffering from NSCLC and esophageal carcinoma. Increase in DKK1 levels was also associated with resistance to platina-based chemotherapy [174]. Based on previous research data, the role of WNT signaling in ABC transporter regulation appears contradictory, therefore more studies are needed to understand the molecular regulation and to identify potential therapeutic targets in WNT pathway associated chemoresistance.

Although increased expression of drug transporters in chemoresistant lung tumors suggests that the drug transporters might be useful therapeutic targets, sadly, clinical trials using ABC transporter inhibitors have not been successful. For example, the ABCB1 (MDR1) inhibitors tariquidar and CBT-1(R) [175–177] were tested unsuccessfully in two phase III clinical studies where treatment of stage IIIB/IV NSCLC patients with tariquidar in combination with vinorelbine or carboplatin/paclitaxel did not show any advantages and the trial was finished prematurely (ID NCT00042302 and NCT00042315) [178]. Additionally, inhibitors of efflux transporters can seriously damage the non-cancerous stem cell pool by increasing toxicity and enhancing the serious side effects of such therapies [179].

## Conclusions

Complex deregulation of WNT signaling is an important element of lung carcinogenesis, controlling not just the carcinogenic process, but also tumor vascularization, drug response and disease progression. While current cancer research frequently cites the importance of precision medicine, therapeutic approaches and even drug development are still strongly focused on genetic mutations [180]. Individual variations in genetic driver and passenger mutations, along with non-mutated but deregulated signaling pathway combinations receive less attention, but can be just as important. Novel drugs are therefore under development to interfere with molecules of the WNT signaling pathway. Recently, NSCLC patients have been recruited into a clinical trial (NCT01957007) testing an anti-FZD7 antibody (OMP18R5 or Vantictumab) in combination therapy [181]. Further studies, however, are also needed to show the effect of WNT pathway interference on tumor cells and non-tumor cells alike [182]. The WNT-receiving cells respond to WNT proteins in a concentration-dependent manner by activating different target genes. Simply, blocking a receptor therefore may result in different cellular response in tumor cells and non-tumor cells at various points of the WNT ligand concentration gradient. To understand such complexity, experimental models of human cancer tissues [183] are essential for future studies. This will allow us to expand our understanding of carcinogenesis beyond mutation analysis and allow intricate investigation of the tissue microenvironment and its effect on epigenetic signal modulation in carcinogenesis and therapeutic drug response.

## Abbreviations

ABC: ATP-binding cassette
ABCB1: ATP-binding cassette transporter protein B1
ABCG2: ATP-binding cassette transporter protein G2
AC: adenocarcinoma
AE: alveolar epithelium
AEC: alveolar epithelial cell
ALK: anaplastic limphoma kinase
AP: activator protein
APC: adenomatous polyposis coli
AT: alveolar type
AXIN: Axis inhibition protein
CaMKII: calcium calmodulin mediated kinase
CBP: CREB-binding protein
CDX: caudal type homeobox
CHI3L3: chitinase-3 like 3
CREB: cAMP response element-binding protein
CSC: cancer stem cell
DAAM: disheveled associated activator of morphogenesis
DACT: Dapper homolog
DKK: Dickkopf-related protein
DNMT3A/3B: DNA methyltransferase 3 alpha/ 3 beta
DVL: Disheveled
E-cadherin: epithelial cadherin
EGFR: epidermal growth factor receptor
EMT: epithelial-mesenchymal transformation
EMX: empty spiracles homeobox
EpCAM: epithelial cell adhesion molecule
FGF: fibroblast growth factor
FLOT: flotillin
FRP: Frizzled-related protein
FZD: Frizzled receptor
GPC: glypican
GSK-3-beta: glycogen synthase kinase 3-beta
HDPR: human homolog of Dapper
HIF1alpha: hypoxia-inducible factor 1 alpha
HNF4A: hepatocyte nuclear factor 4 alpha
IGF: insulin-like growth factor
ING: inhibitor of growth family
JNK: Jun N-terminal kinase
LC: lung cancer
LCC: large cell carcinoma
LEF: lymphoid enhancer-binding factor
LGR: leucin-rich repeat containing G-protein coupled receptor
LRP: lipoprotein receptor-related protein
miRNA: microRNA
MMP: matrix metalloproteinase
N-cadherin: neural cadherin
NFAT: nuclear factor of activated T-cell
NKD: naked cuticle homolog
NLK: nemo-like kinase
NSCLC: non-small cell lung cancer
OCT: octamer-binding transcription factor
PCP: planar cell polarity
PE: primordial epithelium
PKC: protein kinase C
PNEC: pulmonary neuroendocrine cell
PPAR: peroxisome proliferator activated receptor
RAC1: Ras-related C3 botulinum toxin substrate 1
RAGE: receptor for advanced glycation end-product
Rb: retinoblastoma protein
RhoA: Ras homologue gene family member A
ROCK: Rhos-associated coiled-coil-containing protein kinase 1
ROR2: receptor tyrosine kinase-like orphan receptor
RUNX: runt-related transcription factor
RXR: retionid X receptor
RYK: receptor-like tyrosine kinase
SCC: squamous cell carcinoma
SCLC: small cell lung cancer
SHH: sonic hedgehog
SLC: solute carrier transporter
STAT: signal transducer and activator of transcription
TAK: transforming growth factor beta-activated kinase
TCF: T-cell factor
TGFbeta: transforming growth factor beta
TMEM: transmembrane protein
VE-cadherin: vascular endothelial cadherin
VEGF-A: vascular endothelial growth factor A
VEGFR: vascular endothelial growth factor receptor
WIF: WNT inhibitory factor
WNT: wingless/int
